# SimpLiFiCPM: A Simple and Lightweight Filter-Based Algorithm for Circular Pattern Matching

**DOI:** 10.1155/2015/259320

**Published:** 2015-10-18

**Authors:** Md. Aashikur Rahman Azim, Costas S. Iliopoulos, M. Sohel Rahman, M. Samiruzzaman

**Affiliations:** ^1^A*ℓ*EDA Group, Department of CSE, Bangladesh University of Engineering & Technology, Dhaka 1215, Bangladesh; ^2^Department of Informatics, King's College London, Strand, London WC2R 2LS, UK

## Abstract

This paper deals with the circular pattern matching (CPM) problem, which appears as an interesting problem in many biological contexts. CPM consists in finding all occurrences of the rotations of a pattern *𝒫* of length *m* in a text *𝒯* of length *n*. In this paper, we present SimpLiFiCPM (pronounced “Simplify CPM”), a simple and lightweight filter-based algorithm to solve the problem. We compare our algorithm with the state-of-the-art algorithms and the results are found to be excellent. Much of the speed of our algorithm comes from the fact that our filters are effective but extremely simple and lightweight.

## 1. Introduction

The classical pattern matching problem is to find all the occurrences of a given pattern *𝒫* of length *m* in a text *𝒯* of length *n*, both being sequences of characters drawn from a finite character set Σ. This problem is interesting as a fundamental computer science problem and is a basic requirement of many practical applications. The circular pattern, denoted by *𝒞*(*𝒫*), corresponding to a given pattern *𝒫* = *𝒫*_1_ ⋯ *𝒫*_*m*_, is formed by connecting *𝒫*_1_ with *𝒫*_*m*_ and forming a sort of a cycle; this gives us the notion where the same circular pattern can be seen as *m* different linear patterns, which would all be considered equivalent. In the circular pattern matching (CPM) problem, we are interested in pattern matching between the text *𝒯* and the circular pattern *𝒞*(*𝒫*) of a given pattern *𝒫*. We can view *𝒞*(*𝒫*) as a set of *m* patterns starting at positions *j* ∈ [1 : *m*] and wrapping around the end. In other words, in CPM, we search for all “conjugates” (two words *x*, *y* are conjugate if there exist words *u*, *v* such that *x* = *uv* and *y* = *vu*) of a given pattern in a given text.

The problem of circular pattern matching has been considered in [1], where an *𝒪*(*n*)-time algorithm is presented. A naive solution with quadratic complexity consists in applying a classical algorithm for searching a finite set of strings after having built the* trie* of rotations of *𝒫*. The approach presented in [1] consists in preprocessing *𝒫* by constructing a suffix automaton of the string *𝒫𝒫*, by noting that every rotation of *𝒫* is a factor of *𝒫𝒫*. Then, by feeding *𝒯* into the automaton, the lengths of the longest factors of *𝒫𝒫* occurring in *𝒯* can be found by the links followed in the automaton in time *𝒪*(*n*). In [2], the authors have presented an optimal average-case algorithm for CPM, by also showing that the average-case lower bound for the (linear) pattern matching of *𝒪*(*n* log⁡_*σ*_ *m*/*m*) also holds for CPM, where *σ* = |Σ|. Recently, in [3], the authors have presented two fast average-case algorithms based on word-level parallelism. The first algorithm requires average-case time *𝒪*(*n* log⁡_*σ*_ *m*/*w*), where *w* is the number of bits in the computer word. The second one is based on a mixture of word-level parallelism and *q*-grams. The authors have shown that with the addition of *q*-grams, and by setting *q* = *𝒪*(log⁡_*σ*_ *m*), an optimal average-case time of *𝒪*(*n* log⁡_*σ*_ *m*/*m*) can be achieved. Very recently in [4], the authors have presented an efficient algorithm for CPM that runs in *𝒪*(*n*) time on average. To the best of our knowledge, this is the fastest running algorithm for CPM in practice to date.

Notably, indexing circular patterns [5] and variations of approximate circular pattern matching under the edit distance model [6] have also been considered in the literature. Approximate circular pattern matching has also been studied recently in [4, 7]. In this paper however, we focus on the exact version of CPM.

Apart from being interesting from the pure combinatorial point of view, CPM has applications in areas like geometry, astronomy, computational biology, and so forth. For example, the following application in geometry was discussed in [5]. A polygon may be encoded spelling its coordinates. Now, given the data stream of a number of polygons, we may need to find out whether a desired polygon exists in the data stream. The difficulty in this situation lies in the fact that the same polygon may be encoded differently depending on its “starting” coordinate and hence, there exist *k* possible encodings where *k* is the number of vertices of the polygon. Therefore, instead of traditional pattern matching, we need to resort to problem CPM. This problem seems to be useful in computer graphics as well and hence may be used as a built-in function in graphics cards handling polygon rendering.

CPM in fact appears in many biological contexts. This type of circular pattern occurs in the DNA of viruses [9, 10], bacteria [11], eukaryotic cells [12], and archaea [13]. As a result, as has been noted in [14], algorithms on circular strings seem to be important in the analysis of organisms with such structures. Circular strings have also been studied in the context of sequence alignment. In [15], basic algorithms for pairwise and multiple circular sequence alignment have been presented. These results have later been improved in [16], where an additional preprocessing stage is added to speed up the execution time of the algorithm. In [17], the authors also have presented efficient algorithms for finding the optimal alignment and consensus sequence of circular sequences under the Hamming distance metric.

Furthermore, as has been mentioned in [5], this problem seems to be related to the much studied swap matching problem (in CPM, the patterns can be thought of as having a swap of two parts of it) [18] and also to the problem of pattern matching with address error (the circular pattern can be thought of as having a special type of address error) [19, 20]. For further details on the motivation and applications of this problem in computational biology and other areas the readers are kindly referred to [9–17] and references therein.

In this paper, we present SimpLiFiCPM (pronounced Simplify CPM), which is a fast and efficient algorithm for the circular pattern matching problem based on some filtering techniques. In particular, we employ a number of simple and effective filters to preprocess the given pattern and the text. After this preprocessing, we get a text of reduced length on which we can apply any existing state-of-the-art algorithms to get the occurrences of the circular pattern. So, as the name sounds, SimpLiFiCPM, in some sense, simplifies the search space of the circular pattern matching problem.

We have conducted extensive experiments to compare our algorithm with the state-of-the-art algorithms and the results are found to be excellent. Our algorithm turns out to be much faster in practice because of the huge reduction in the search space through filtering. Also, the filtering techniques we use are simple and lightweight but as can be realized from the results extremely effective.

The rest of the paper is organized as follows.  Section 2 gives a preliminary description of some terminologies and concepts related to stringology that will be used throughout this paper. In Section 3 we describe our filtering algorithms.  Section 4 presents the experimental results.  Section 5 draws conclusion followed by some future research directions.

## 2. Preliminaries

Let Σ be a finite* alphabet*. An element of Σ^∗^ is called a* string*. The length of a string *w* is denoted by |*w*|. The empty string *ϵ* is a string of length 0; that is, |*ϵ*| = 0. Let Σ^+^ = Σ^∗^ − {*ϵ*}. For a string *w* = *xyz*, *x*, *y*, and *z* are called a* prefix*,* factor* (or, equivalently,* substring*), and* suffix* of *w*, respectively. The *i*th character of a string *w* is denoted by *w*[*i*] for 1 ≤ *i* ≤ |*w*|, and the factor of a string *w* that begins at position *i* and ends at position *j* is denoted by *w*[*i* : *j*] for 1 ≤ *i* ≤ *j* ≤ |*w*|. For convenience, we assume *w*[*i* : *j*] = *ϵ* if *j* < *i*. A *k*-factor is a factor of length *k*.

A circular string of length *m* can be viewed as a traditional linear string which has the leftmost and rightmost symbols wrapped around and stuck together in some way. Under this notion, the same circular string can be seen as *m* different linear strings, which would all be considered equivalent. Given a string *𝒫* of length *m*, we denote by *𝒫* ^*i*^ = *𝒫*[*i* : *m*]*𝒫*[1 : *i* − 1], 0 < *i* < *m*, the *i*th* rotation* of *𝒫* and *𝒫* ^0^ = *𝒫*.

**Example 1 .**

Suppose we have a pattern *𝒫* = *atcgatg*. The pattern *𝒫* has the following rotations (i.e., conjugates): *𝒫* ^1^ = *tcgatga*,  *𝒫* ^2^ = *cgatgat*,  *𝒫* ^3^ = *gatgatc*,  *𝒫* ^4^ = *atgatcg*,  *𝒫* ^5^ = *tgatcga*, and  *𝒫* ^6^ = *gatcgat*.

Here we consider the problem of finding occurrences of a pattern string *𝒫* of length *m* with circular structure in a text string *𝒯* of length *n* with linear structure. For instance, the DNA sequence of many viruses has a circular structure. So if a biologist wishes to find occurrences of a particular virus in a carrier's DNA sequence, which may not be circular, (s)he must locate all positions in *𝒯* where at least one rotation of *𝒫* occurs. This is the problem of* circular pattern matching* (CPM).

We consider the DNA alphabet, that is, Σ = {*a*, *c*, *g*, *t*}. In our approach, each character of the alphabet is associated with a numeric value as follows. Each character is assigned a unique number from the range [1 ⋯ |Σ|]. Although this is not essential, we conveniently assign the numbers from the range [1 ⋯ |Σ|] to the characters of Σ following their inherent lexicographical order. We use *num*(*x*), *x* ∈ Σ, to denote the numeric value of the character *x*. So, we have *num*(*a*) = 1, *num*(*c*) = 2, *num*(*g*) = 3, and *num*(*t*) = 4. For a string *S*, we use the notation *S*_*N*_ to denote the numeric representation of the string *S*; *S*_*N*_[*i*] denotes the numeric value of the character *S*[*i*]. So, if *S*[*i*] = *g* then *S*_*N*_[*i*] = *num*(*g*) = 3. The concept of circular strings and their rotations also applies naturally on their numeric representations as is illustrated in Example 2 below.

**Example 2 .**

Suppose we have a pattern *𝒫* = *atcgatg*. The numeric representation of *𝒫* is *𝒫*_*N*_ = 1423143. And this numeric representation has the following rotations: *𝒫*_*N*_^1^ = 4231431, *𝒫*_*N*_^2^ = 2314314, *𝒫*_*N*_^3^ = 3143142, *𝒫*_*N*_^4^ = 1431423, *𝒫*_*N*_^5^ = 4314231, and *𝒫*_*N*_^6^ = 3142314.

The problem we handle in this paper can be formally defined as follows.

**Problem 3 (circular pattern matching (CPM)).**

Given a pattern *𝒫* of length *m* and a text *𝒯* of length *n* ≥ *m*, find all factors *ℱ* of *𝒯* such that *ℱ* = *𝒫* ^*i*^, for some 0 ≤ *i* < *m*. And if we have *ℱ* = *𝒫* ^*i*^ for some 0 ≤ *i* < *m*, then we say that the circular pattern *𝒞*(*𝒫*) matches *𝒯* at position *i*.

In the context of our filter-based algorithm the concept of false positives and negatives is important. So, we briefly discuss this concept here. Suppose we have an algorithm *𝒜* to solve a problem *ℬ*. Now suppose that *𝒮*_true_ represents the set of true solutions for problem *ℬ*. Further suppose that *𝒜* computes the set *𝒮*_*𝒜*_ as the set of solutions for *ℬ*. Now assume that *𝒮*_true_ ≠ *𝒮*_*𝒜*_. Then, the set of false positives can be computed as follows: *𝒮*_*𝒜*_∖*𝒮*_true_, where “∖” refers to the set difference operation. In other words, the set computed by *𝒜* contains some solutions that are not true solutions for problem *ℬ*. And these are the false positives, because *𝒮*_*𝒜*_ falsely marked these as solutions (i.e., positive). On the other hand, the set of false negatives can be computed as follows: *𝒮*_true_∖*𝒮*_*𝒜*_. In other words, false negatives are those members in *𝒮*_true_ that are absent in *𝒮*_*𝒜*_. These are false negatives because *𝒮*_*𝒜*_ falsely marked these as nonsolutions (i.e., negative).

## 3. Our Approach

As has been mentioned above, our algorithm is based on some filtering techniques. Suppose we are given a pattern *𝒫* and a text *𝒯*. We will frequently and conveniently use the expression “*𝒞*(*𝒫*) matches *𝒯* at position *i*” (or, equivalently, “*𝒫* circularly matches *𝒯* at position *i*”) to indicate that one of the conjugates of *𝒫* matches *𝒯* at position *i*. We start with a brief overview of our approach below.

### 3.1. Overview of SimpLiFiCPM

In SimpLiFiCPM, we first employ a number of filters to compute a set *𝒩* of indexes of *𝒯* such that *𝒞*(*𝒫*) matches *𝒯* at position *i* ∈ *𝒩*. As will be clear shortly, our filters are unable to compute the true set of indexes and hence *𝒩* may have false positives. However, our filters are designed in such a way that there are no false negatives. Hence, for all *j* ∉ *𝒩*, we can be sure that there is no match. On the other hand, for all *i* ∈ *𝒩*, we may or may not have a match; that is, we may have false positives. So, after we have computed *𝒩*, we compute *𝒯*′, a reduced version of *𝒯* concatenating all the factors *ℱ*[*i* ⋯ *i* + *m* − 1], *i* ∈ *𝒩*, putting a special character $ ∉ Σ in between the factors. One essential detail is as follows. There can be *i*, *j* ∈ *𝒩* such that 1 < *j* − *i* < *p*. In other words, there can exist overlapping factors matching with *𝒞*(*P*). However, this can be handled easily through simple bookkeeping as will be evident from our algorithm in later sections. Clearly, once we have computed the reduced text *𝒯*′ we can employ any state-of-the-art algorithm to solve CPM on *𝒯*′ to get the actual occurrences. So the most essential and useful feature of SimpLiFiCPM is the application of filters to get a reduced text on which any existing algorithm can be applied to solve CPM.

### 3.2. Filters of SimpLiFiCPM

In SimpLiFiCPM, we employ 6 filters. In this section we describe these filters. We also discuss the related notions and notations needed to describe these filters. In what follows we describe our filters in the context of two strings of equal length *n*, namely, *𝒫* and *𝒯*, where the former is a circular string and the latter is linear. We will devise and apply different functions on these strings and present observations related to these functions which in the sequel will lead us to our desired filter. The key to our observations and the resulting filters is the fact that each function we devise results in a unique output when applied to the rotations of a circular string. For example, consider a hypothetical function *𝒳*. We will always have the relation that *𝒳*(*𝒫*) = *𝒳*(*𝒫* ^*i*^) for all 1 ≤ *i* < *n*. Recall that *𝒫* ^0^ actually denotes *𝒫*. For the sake of conciseness, for such functions, we will abuse the notation a bit and use *𝒳*(*𝒞*(*𝒫*)) to represent *𝒳*(*𝒫* ^*i*^) for all 0 ≤ *i* < |*𝒫*|.

#### 3.2.1. Filter 1

We define the function *sum* on a string *𝒫* of length *m* as follows: *sum*(*𝒫*) = ∑_*i*=1_^*m*^ *P*_*N*_[*i*]. Our first filter, Filter 1, is based on this *sum* function. We have the following observation.

**Observation 1 .**

Consider a circular string *𝒫* and a linear string *𝒯* both having length *n*. If *𝒞*(*𝒫*) matches *𝒯*, then we must have *sum*(*𝒞*(*𝒫*)) = *sum*(*𝒯*).

**Example 4 .**

Consider *𝒫* = *atcgatg𝒯* = *tgatcga*. As can be easily verified, here *𝒫* circularly matches *𝒯*. In fact the match is due to the conjugate *𝒫* ^5^. Now we have *𝒯*_*N*_ = 4314231 and *sum*(*𝒯*) = 18. Then, according to Observation 1, we must have *sum*(*𝒞*(*𝒫*)) = 18. This can indeed be verified easily.

Now consider another string *𝒯*′ = *atagctg*, which is slightly different from *𝒯*. It can be easily verified that *𝒞*(*𝒫*) does not match *𝒯*′. Now, *𝒯*_*N*_′ = 1413243 and hence here also we have *sum*(*𝒯*′) = 18 = *sum*(*𝒞*(*𝒫*)). This is an example of a false positive with respect to Filter 1.

#### 3.2.2. Filters 2 and 3

Our second and third filters, that is, Filters 2 and 3, depend on a notion of distance between consecutive characters of a string. The* distance* between two consecutive characters of a string *𝒫* of length *m* is defined by *distance*(*𝒫*[*i*], *𝒫*[*i* + 1]) = *𝒫*_*𝒩*_[*i*] − *𝒫*_*𝒩*_[*i* + 1], where 1 ≤ *i* ≤ *m* − 1. We define *total*_*distance*(*P*) = ∑_*i*=1_^*m*−1^ *distance*(*𝒫*[*i*], *𝒫*[*i* + 1]). We also define an absolute version of it: *abs*_*total*_*distance*(*P*) = ∑_*i*=1_^*m*−1^ *abs*(*distance*(*𝒫*[*i*], *𝒫*[*i* + 1])), where *abs*(*x*) returns the magnitude of *x* ignoring the sign. Before we apply these two functions on our strings to get our filters, we need to do a simple preprocessing on the respective string, that is, *𝒫* in this case as follows. We extend the string *𝒫* by concatenating the first character of *𝒫* at its end. We use *ext*(*𝒫*) to denote the resultant string. So, we have *ext*(*𝒫*) = *𝒫𝒫*[1]. Since *ext*(*𝒫*) can simply be treated as another string, we can easily extend the notation and concept of *𝒞*(*𝒫*) over *ext*(*𝒫*) and we continue to abuse the notation a bit for the sake of conciseness as mentioned at the beginning of Section 3.2 (just before Section 3.2.1).

Now we have the following observation which is the basis of our Filter 2.

**Observation 2 .**

Consider a circular string *𝒫* and a linear string *𝒯* both having length *n* and assume that *𝒜* = *ext*(*𝒫*) and *ℬ* = *ext*(*𝒯*). If *𝒞*(*𝒫*) matches *𝒯*, then, we must have *abs*_*total*_*distance*(*𝒞*(*𝒜*)) = *abs*_*total*_*distance*(*ℬ*). Note carefully that the function *abs*_*total*_*distance*() has been applied on the extended strings.

**Example 5 .**

Consider the same two strings of Example 4, that is, *𝒫* = *atcgatg𝒯* = *tgatcga*. Here *𝒫* circularly matches *𝒯* (due to the conjugate *𝒫* ^5^). Now consider the extended strings and assume that *𝒜* = *ext*(*𝒫*) and *ℬ* = *ext*(*𝒯*). We have *𝒯*_*N*_ = 4314231. Hence *ℬ*_*N*_ = 43142314. Hence, *abs*_*total*_*distance*(*ℬ*) = 14. It can be easily verified that *abs*_*total*_*distance*(*𝒞*(*𝒜*)) is also 14.

Now consider another string *𝒯*′ = *atagctg* of the same length, which is slightly different from *𝒯*. It can easily be checked that *𝒞*(*𝒫*) does not match *𝒯*′. However, assuming that *ℬ*′ = *ext*(*𝒯*′) we find that *abs*_*total*_*distance*(*ℬ*′) is still 14. So, this is an example of a false positive with respect to Filter 2.

Now we present the following related observation which is the basis of our Filter 3. Note that Observation 2 differs with Observation 3 only through using the absolute version of the function used in the latter.

**Observation 3 .**

Consider a circular string *𝒫* and a linear string *𝒯* both having length *n* and assume that *𝒜* = *ext*(*𝒫*) and *ℬ* = *ext*(*𝒯*). If *𝒞*(*𝒫*) matches *𝒯*, then, we must have *total*_*distance*(*𝒞*(*𝒜*)) = *total*_*distance*(*ℬ*). Note carefully that the function *total*_*distance*() has been applied on the extended strings.

**Example 6 .**

Consider the same two strings of previous examples, that is, *𝒫* = *atcgatg𝒯* = *tgatcga*. Here *𝒫* circularly matches *𝒯* (due to the conjugate *𝒫* ^5^). Now consider the extended strings and assume that *𝒜* = *ext*(*𝒫*) and *ℬ* = *ext*(*𝒯*). We have *𝒯*_*N*_ = 4314231. Hence *ℬ*_*N*_ = 43142314. Hence, *total*_*distance*(*ℬ*) = 0. It can be easily verified that *total*_*distance*(*𝒞*(*𝒜*)) is also 0.

Now consider another string *𝒯*′ = *atagctg* of the same length, which is slightly different from *𝒯*. It can easily be checked that *𝒞*(*𝒫*) does not match *𝒯*′. However, assuming that *ℬ*′ = *ext*(*𝒯*′) we find that *total*_*distance*(*ℬ*′) is still 0. So, this is an example of a false positive with respect to Filter 3.

#### 3.2.3. Filter 4

Filter 4 uses the *sum*() function used by Filter 1, albeit in a slightly different way. In particular, it applies the *sum*() function on individual characters. So, for *x* ∈ Σ we define *sum*_*x*_(*𝒫*) = ∑_1≤*i*≤|*𝒫*|,*𝒫*[*i*]=*x*_ *P*_*N*_[*i*]. Now we have the following observation.

**Observation 4 .**

Consider a circular string *𝒫* and a linear string *𝒯* both having length *n*. If *𝒞*(*𝒫*) matches *𝒯*, then, we must have *sum*_*x*_(*𝒞*(*𝒫*)) = *sum*_*x*_(*𝒯*) for all *x* ∈ Σ.

**Example 7 .**

Consider the same two strings of previous examples, that is, *𝒫* = *atcgatg𝒯* = *tgatcga*. Recall that *𝒫* circularly matches *𝒯* (due to the conjugate *𝒫* ^5^). It is easy to calculate that *sum*_*a*_(*𝒯*) = 2, *sum*_*c*_(*𝒯*) = 2, *sum*_*g*_(*𝒯*) = 6, and *sum*_*t*_(*𝒯*) = 8. Hence according to Observation 4, the individual sum values for all the conjugates of *𝒫* must also match this. It can be easily verified that this is indeed the case.

Now consider the other string *𝒯*′ = *atagctg* of the same length, which is slightly different from *𝒯*. It can easily be checked that *𝒞*(*𝒫*) does not match *𝒯*′. However, as we can see, still we have *sum*_*a*_(*𝒯*′) = 2, *sum*_*c*_(*𝒯*′) = 2, *sum*_*g*_(*𝒯*′) = 6, and *sum*_*t*_(*𝒯*′) = 8. This is an example of a false positive with respect to Filter 4.

Notably, a similar idea has been used by Kahveci et al. in [21] for indexing large strings with a goal to achieve fast local alignment of large genomes. In particular, for a DNA string, Kahveci et al. compute the so-called* frequency vector* that keeps track of the frequency of each character of the DNA alphabet in the string.

#### 3.2.4. Filter 5

Filter 5 depends on modulo operation between two consecutive characters. A modulo operation between two consecutive characters of a string *𝒫* of length *m* is defined as follows: *modulo*(*𝒫*[*i*], *𝒫*[*i* + 1]) = *𝒫*_*N*_[*i*]%*𝒫*_*N*_[*i* + 1], where 1 ≤ *i* ≤ *m* − 1. We define *sum*_*modulo*(*𝒫*) to be the summation of the results of the modulo operations on the consecutive characters of *𝒫*. More formally, *sum*_*modulo*(*P*) = ∑_*i*=1_^*m*−1^ *modulo*(*𝒫*[*i*], *𝒫*[*i* + 1]). Now we present the following observation which is the basis of Filter 5. Note that this observation is applied on the extended versions of the respective strings.

**Observation 5 .**

Consider a circular string *𝒫* and a linear string *𝒯* both having length *n* and assume that *𝒜* = *ext*(*𝒫*) and *ℬ* = *ext*(*𝒯*). If *𝒞*(*𝒫*) matches *𝒯*, then, we must have *sum*_*modulo*(*𝒞*(*𝒜*)) = *sum*_*modulo*(*ℬ*). Note carefully that the function *sum*_*modulo*() has been applied on the extended strings.

**Example 8 .**

Consider the same two strings of previous examples, that is, *𝒫* = *atcgatg𝒯* = *tgatcga*. Recall that *𝒫* circularly matches *𝒯* (due to the conjugate *𝒫* ^5^). Now consider the extended strings and assume that *𝒜* = *ext*(*𝒫*) and *ℬ* = *ext*(*𝒯*). We have *𝒯*_*N*_ = 4314231. Hence *ℬ*_*N*_ = 43142314. Hence, *sum*_*modulo*(*ℬ*) = 5. Now according to Observation 5, we must also have *sum*_*modulo*(*𝒞*(*𝒜*)) = 5. This is indeed true.

Now consider another string *𝒯*′ = *tgagatc* of the same length, which is different from *𝒯*. It can easily be checked that *𝒞*(*𝒫*) does not match *𝒯*′. However, assuming that *ℬ*′ = *ext*(*𝒯*′) we find that *sum*_*modulo*(*ℬ*′) is still 5. So, this is an example of a false positive with respect to Filter 5.

#### 3.2.5. Filter 6

In Filter 6 we employ the *xor*() operation. A bitwise exclusive-OR (*xor*()) operation between two consecutive characters of a string *𝒫* of length *m* is defined as follows: *xor*(*𝒫*[*i*], *𝒫*[*i* + 1]) = *𝒫*_*N*_[*i*]∧*𝒫*_*N*_[*i* + 1], where 1 ≤ *i* ≤ *m* − 1. We define *sum*_*xor*(*𝒫*) to be the summation of the results of the xor operations on the consecutive characters of *𝒫*. More formally, *sum*_*xor*(*P*) = ∑_*i*=1_^*m*−1^ *xor*(*𝒫*[*i*], *𝒫*[*i* + 1]). Now we present the following observation which is the basis of Filter 6. Note that this observation is applied on the extended versions of the respective strings.

**Observation 6 .**

Consider a circular string *𝒫* and a linear string *𝒯* both having length *n* and assume that *𝒜* = *ext*(*𝒫*) and *ℬ* = *ext*(*𝒯*). If *𝒞*(*𝒫*) matches *𝒯*, then, we must have *sum*_*xor*(*𝒞*(*𝒜*)) = *sum*_*xor*(*ℬ*). Note carefully that the function *sum*_*xor*() has been applied on the extended strings.

**Example 9 .**

Consider the same two strings of previous examples, that is, *𝒫* = *atcgatg𝒯* = *tgatcga*. Recall that *𝒫* circularly matches *𝒯* (due to the conjugate *𝒫* ^5^). Now consider the extended strings and assume that *𝒜* = *ext*(*𝒫*) and *ℬ* = *ext*(*𝒯*). We have *𝒯*_*N*_ = 4314231. Hence *ℬ*_*N*_ = 43142314. Hence, *sum*_*xor*(*ℬ*) = 28. Now according to Observation 5, we must also have *sum*_*x*_ *or*(*𝒞*(*𝒜*)) = 28. As can be verified easily, this is indeed the case.

Now consider another string *𝒯*′ = *gt*a*gatc* of the same length, which is different from *𝒯*. It can easily be checked that *𝒞*(*𝒫*) does not match *𝒯*′. However, assuming that *ℬ*′ = *ext*(*𝒯*′) we find that *sum*_*xor*(*ℬ*′) is still 28. So, this is an example of a false positive with respect to Filter 5.

#### 3.2.6. Discussion with respect to [8]

At this point a brief discussion with respect to our preliminary work in [8] is in order. To reduce the text *𝒯*, we also employed six filters in [8]. While Filter 1 and Filter 4 remain identical, in SimpLiFiCPM, we have changed and improved Filters 2, 3, 5, and 6 to get better results. In particular, we have introduced the concept of extended string here and modified the filters accordingly. Much of the efficiency of these new filters comes from the fact that in the preliminary version, without the extended strings, we had to deal with a set of values as the output of the functions creating a small bottleneck. On the contrary, SimpLiFiCPM now needs to deal with only one value as the output of the functions of Filters 2, 3, 5, and 6. This makes SimpLiFiCPM even faster than its predecessor. This is evident from the experimental results presented later. Notably, this has essentially brought some more changes in the overall algorithm. In particular in the searching phase of the algorithm we now need to adapt accordingly to apply the corresponding filters on the extended strings. But the overall improvement outweighs this extra work by a long margin.

### 3.3. Circular Pattern Signature Using the Filters

In this section, we discuss an *𝒪*(*m*)-time algorithm that SimpLiFiCPM uses to compute the signature of the circular pattern *𝒞*(*𝒫*) corresponding to pattern *𝒫* of length *m*. This signature is used at a later stage to filter the text. Here, we need five variables to save the output of the functions used for Filters 1, 2, 3, 5, and 6 (based on Observations 1, 2, 3, 5, and 6). And we need a list of size 4 to save the values of the function used in Filter 4 (Observation 4). We start with the extended string *ext*(*𝒫*) = *𝒫*[1 : *m*]*𝒫*[1] and compute the values according to Observations 1 to 6. The algorithm will iterate *m* + 1 times and hence the overall runtime of the algorithm is *𝒪*(*m*). The algorithm is presented in Procedure *ECPS*_*FT* (Algorithm 1).

### 3.4. Reduction of Search Space in the Text

Now we present an *𝒪*(*n*) runtime algorithm that SimpLiFiCPM uses to reduce the search space of the text applying the six filters presented above. It takes as input the pattern *𝒫*[1 : *m*] of length *m* and the text *𝒯*[1 : *n*] of length *n*. It calls Procedure *ECPS*_*FT* with *𝒫*[1 : *m*] as parameter and uses the output. It then applies the same technique that is applied in Procedure *ECPS*_*FT* (Algorithm 1). We apply a sliding window approach with window length of *m* and calculate the values applying the functions according to Observations 1–6 on the factor of *𝒯* captured by the window. Note that, for Observations 2, 3, 5, and 6, we need to consider the extended string and hence the factor of *𝒯* within the window need be extended accordingly for calculating the values. After we calculate the values for a factor of *𝒯*, we check it against the returned values of Procedure *ECPS*_*FT*. If it matches, then we output the factor to a file. Note that, in case of overlapping factors (e.g., when the consecutive windows need to output the factors to a file), Procedure *ECPS*_*FT* outputs only the nonoverlapped characters. And Procedure *ECPS*_*FT* uses a $ marker to mark the boundaries of nonconsecutive factors, where $ ∉ Σ.

Now note that we can compute the values of consecutive factors of *𝒯* using the sliding window approach quite efficiently as follows. For the first factor, that is, *𝒯*[1 ⋯ *m*], we exactly follow the strategy of Procedure *ECPS*_*FT*. When it is done, we slide the window by one character and we only need to remove the contribution of the leftmost character of the previous window and add the contribution of the rightmost character of the new window. The functions are such that this can be done very easily using simple constant time operations. The only other issue that needs to be taken care of is due to the use of the extended string in four of the filters. But this too does not need more than simple constant time operations. Therefore, overall runtime of the algorithm is *𝒪*(*m*) + *𝒪*(*n* − *m*) = *𝒪*(*n*). The algorithm is presented in the form of Procedure *RSS*_*FT* (Algorithm 2).

### 3.5. The Combined SimpLiFiCPM Algorithm

In this section we combine the algorithms presented so far and present the complete view of SimpLiFiCPM. We have already described the two main components of SimpLiFiCPM, namely, Procedure *ECPS*_*FT* and Procedure *RSS*_*FT*, that in fact calls the former. Now Procedure *RSS*_*FT* provides a reduced text *𝒯*′ (say) after filtering. At this point SimpLiFiCPM can use any algorithm that can solve CPM and apply it over *𝒯*′ and output the occurrences. Now, suppose SimpLiFiCPM uses algorithm *𝒜* at this stage which runs in *𝒪*(*f*(|*𝒯*′|)) time. Then, clearly, the overall running time of SimpLiFiCPM is *𝒪*(*n*) + *𝒪*(*f*(|*𝒯*′|)). For example, if SimpLiFiCPM uses the linear time algorithm of [1], then clearly the overall theoretical running time of SimpLiFiCPM will be *𝒪*(*n*).

In our implementation however we have used the recent algorithm of [4], which is a linear time algorithm on average and the fastest algorithm in practice to the best of our knowledge. In particular, in [4], the authors have presented an approximate circular string matching algorithm with *k*-mismatches (ACSMF-Simple) via filtering. They have built a library for ACSMF-Simple algorithm. The library is freely available and can be found in [22]. In this algorithm, if we set *k* = 0, then ACSMF-Simple works for the exact matching case. In what follows, we will refer to this algorithm as ACSMF-SimpleZero*k*. We have implemented SimpLiFiCPM using ACSMF-SimpleZero*k*; that is, we have used ACSMF-Simple algorithm simply by putting *k* = 0.

### 3.6. An Illustrative Example

Now that we have fully described SimpLiFiCPM, in this section we present the simulation of SimpLiFiCPM on a particular example. We only show the simulation up to the output of Procedure *RSS*_*FT*, that is, the output of the reduced text, because afterwards we can employ any state-of-the-art algorithm within SimpLiFiCPM. Consider a pattern *𝒫* = *atcgatg*. The values computed by Procedure *ECPS*_*FT* according to Observations 1 through 6 are as follows, respectively: *local*_*total*_*sum* = 18, *abs*_*sum* = 14, *actual*_*sum* = 0, *local*_*individual*_*sum*[0 : 4] = {2,2, 6,8}, *modulas*_*sum* = 5, and *xor*_*sum* = 28.

Again consider a text string *𝒯* = *tgatcgaaagtaatcgatg*$. For the first sliding window we need to calculate the observation values from *𝒯*[1 : 7]. The observation values according to Procedure *RSS*_*FT* are as follows for *𝒯*[1 : 7]: *local*_*total*_*sum* = 18, *abs*_*sum* = 14, *actual*_*sum* = 0, *local*_*individual*_*sum*[0 : 4] = {2,2, 6,8}, *modulas*_*sum* = 5, and *xor*_*sum* = 28.

The length of *𝒯* is 19. And the length of *𝒫* is 7. So, the algorithm iterates exactly 19 − 7 + 1 = 13 times. Each iteration is illustrated in Table 1.

## 4. Experimental Results

We have implemented SimpLiFiCPM and conducted extensive experiments to analyze its performance. We have coded SimpLiFiCPM in C++ using a GNU compiler with General Public License (GPL). Our code is available at [23]. As has been mentioned already above, our implementation of SimpLiFiCPM uses the ACSMF-SimpleZero*k* [4]. ACSMF-Simple [4] has been implemented as library functions in the C programming language under GNU/Linux operating system. The library implementation is distributed under the GNU General Public License (GPL). It takes as input the pattern *𝒫* of length *m*, the text *𝒯* of length *n*, and the integer threshold *k* < *m* and returns the list of starting positions of the occurrences of the rotations of *𝒫* in *𝒯* with *k*-mismatches as output. In our case we use *k* = 0.

We have used real genome data in our experiments as the text string, *𝒯*. This data has been collected from [24]. Here, we have taken 299 MB of data for our experiments. We have generated random patterns of different length by a random indexing technique in these 299 MB of text string.

We have conducted our experiments on a PowerEdge R820 rack serve PC with 6-core Intel Xeon processor* E5-4600* product family and 64 GB of RAM under GNU/Linux. With the help of the library used in [4], we have compared the running time of our preliminary work in [8] (referred to as Filter-CPM henceforth), ACSMF-SimpleZero*k* of [4], and SimpLiFiCPM.  Table 2 reports the elapsed time and speed-up comparisons for various pattern sizes (500 ≤ *m* ≤ 3000). As can be seen from Table 2, Filter-CPM [8] runs faster than ACSMF-SimpleZero*k* in all cases. And in fact Filter-CPM [8] achieves a minimum of twofold speed-up for all the pattern sizes. Again, referring to the same table, SimpLiFiCPM runs even faster than ACSMF-SimpleZero*k* in all cases. And in fact SimpLiFiCPM achieves a minimum of threefold speed-up for all the pattern sizes.

In order to analyze and understand the effect of our filters we have run a second set of experiments as follows. We have run experiments on three variants of SimpLiFiCPM where the first variant (SimpLiFiCPM-[1 ⋯ 3]) only employs Filters 1 through 3, the second variant (SimpLiFiCPM-[1 ⋯ 4]) only employs Filters 1 through 4, and finally the third variant (SimpLiFiCPM-[1 ⋯ 5]) employs Filters 1 through 5.  Table 2 reports the elapsed time and speed-up comparisons considering various pattern sizes (500 ≤ *m* ≤ 2000) for ACSMF-SimpleZero*k* and the above-mentioned three variants of SimpLiFiCPM. As can be seen from Table 3, ACSMF-SimpleZero*k* is able to beat SimpLiFiCPM-[1 ⋯ 3] in a number of cases. However, SimpLiFiCPM-[1 ⋯ 4] and SimpLiFiCPM-[1 ⋯ 5] significantly run faster than ACSMF-SimpleZero*k* in all cases.

## 5. Conclusions

In this paper, we have employed some effective lightweight filtering technique to reduce the search space of the circular pattern matching (CPM) problem. We have presented SimpLiFiCPM, an extremely fast algorithm based on the above-mentioned filters. We have conducted extensive experimental studies to show the effectiveness of SimpLiFiCPM. In our experiments, SimpLiFiCPM has achieved a minimum of threefold speed-up compared to the state-of-the-art algorithms. Much of the speed of our algorithm comes from the fact that our filters are effective but extremely simple and lightweight. The most intriguing feature of SimpLiFiCPM is perhaps its capability to plug in any algorithm to solve CPM and take advantage of it. We are now working towards adapting the filters so that it could work for the approximate version of CPM.

## Figures and Tables

**Algorithm 1:**
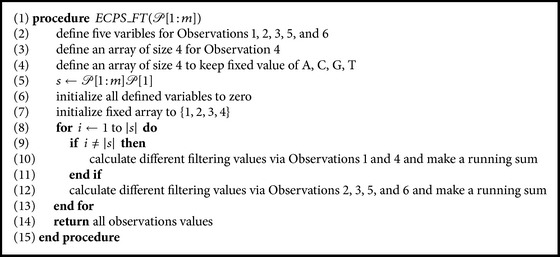
Exact circular pattern signature using Observations 1–6 in a single pass.

**Algorithm 2:**
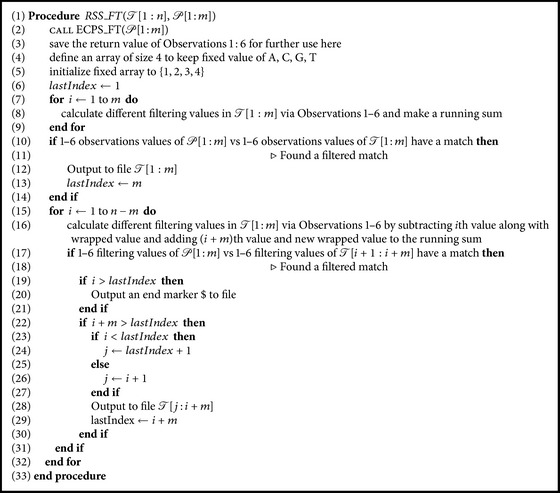
Reduction of search space in a text string using Procedure ECPS_FT.

**Table 1:** An example simulation of SimpLiFiCPM.

Iteration	Local total sum	abs sum	Actual sum	Local individual sum [0 : 4]	modulas sum	xor sum	Does it match with pattern?	Output file
1	18	14	0	{2, 2, 6, 8}	5	28	YES	*tgatcga*
2	15	12	0	{3, 2, 6, 4}	4	18	NO	$
3	13	8	0	{4, 2, 3, 4}	3	14	NO	
4	15	8	0	{3, 2, 6, 4}	6	18	NO	
5	15	8	0	{3, 2, 6, 4}	6	18	NO	
6	14	10	0	{4, 0, 6, 4}	5	18	NO	
7	12	6	0	{5, 0, 3, 4}	4	14	NO	
8	15	12	0	{4, 0, 3, 8}	5	24	NO	
9	16	12	0	{3, 2, 3, 8}	5	28	NO	
10	18	10	0	{2, 2, 6, 8}	6	24	NO	
11	16	14	0	{3, 2, 3, 8}	4	24	NO	
12	16	14	0	{3, 2, 3, 8}	4	24	NO	
13	18	14	0	{2, 2, 6, 8}	5	28	YES	*atcgatg*

**Table 2:** Elapsed time (in seconds) of and speed-up comparisons among Filter-CPM [8], ACSMF-SimpleZero*k* [4], and SimpLiFiCPM on a text of size 299 MB.

*m*	Elapsed time (s) of ACSMF-SimpleZero*k*	Elapsed time (s) of Filter-CPM	Speed-up: ACSMF-SimpleZero*k* versus Filter-CPM	Elapsed time (s) of SimpLiFiCPM	Speed-up: ACSMF-SimpleZero*k* versus SimpLiFiCPM
500	5.938	3.025	2	1.167	5
550	7.914	3.068	3	1.456	5
600	7.691	3.06	3	1.364	6
650	7.836	3.074	3	1.006	8
700	7.739	3.072	3	1.028	8
750	7.82	3.051	3	1.073	7
800	7.839	3.209	2	1.04	8
850	8.382	3.053	3	1.055	8
900	7.646	3.055	3	1.278	6
950	7.876	3.049	3	1.402	6
1000	7.731	3.067	3	1.216	6
1600	7.334	3.206	2	1.182	6
1650	8.239	3.063	3	0.969	9
1700	7.572	3.059	2	1.18	6
1750	5.968	3.066	2	1.144	5
1800	7.551	3.064	2	1.179	6
1850	7.407	3.079	2	1.086	7
1900	7.861	3.225	2	1.126	7
1950	7.339	3.073	2	1.028	7
2000	7.814	3.062	3	1.118	7
2050	5.969	3.057	2	1.988	3
2100	5.173	3.036	2	1.187	4
2150	5.317	3.027	2	1.919	3
2200	6.032	3.168	2	1.927	3
2250	5.009	3.073	2	1.895	3
2300	5.029	3.024	2	1.891	3
2350	5.041	3.047	2	1.887	3
2400	6.036	3.046	2	1.91	3
2450	6.04	3.037	2	1.886	3
2500	7.046	3.029	2	1.976	4
2550	7.042	3.037	2	1.987	4
2600	8.043	4.029	2	2.883	3
2650	8.049	4.03	2	2.884	3
2700	8.031	4.183	2	2.892	3
2750	8.039	4.044	2	2.882	3
2800	9.026	4.067	2	2.886	3
2850	9.154	4.036	2	2.901	3
2900	10.049	4.045	2	3.134	3
2950	11.044	5.052	2	3.876	3
3000	12.044	6.039	2	3.9	3

**Table 3:** Elapsed time (in seconds) of and speed-up comparisons among ACSMF-SimpleZero*k* and three variants of SimpLiFiCPM (considering different combination of the filters) for a text of size 299 MB.

*m*	Filters 1 to 3			Filters 1 to 4			Filters 1 to 5		
	Elapsed time (s) of ACSMF-SimpleZero*k*	Elapsed time (s) of SimpLiFiCPM-[1 ⋯ 3]	Speed-up: ACSMF-SimpleZero*k* versus SimpLiFiCPM-[1 ⋯ 3]	Elapsed time (s) of ACSMF-SimpleZero*k*	Elapsed time (s) of SimpLiFiCPM-[1 ⋯ 4]	Speed-up: ACSMF-SimpleZero*k* versus SimpLiFiCPM-[1 ⋯ 4]	Elapsed time (s) of ACSMF-SimpleZero*k*	Elapsed time (s) of SimpLiFiCPM-[1 ⋯ 5]	Speed-up: ACSMF-SimpleZero*k* versus SimpLiFiCPM-[1 ⋯ 5]
500	6.355	3.522	2	6.373	4.973	1	6.397	2.523	3
550	8.526	20.43	0	8.564	4.866	2	8.38	2.545	3
600	8.149	43.544	0	8.286	4.902	2	8.359	2.518	3
650	8.315	4.35	2	8.448	4.894	2	8.324	2.47	3
700	9.063	7.596	1	8.71	4.9	2	8.249	2.493	3
750	8.399	6.837	1	8.643	5.101	2	8.326	2.478	3
800	8.357	16.293	1	8.346	4.915	2	8.265	2.48	3
850	8.79	10.651	1	8.309	4.924	2	8.48	2.562	3
900	7.959	23.181	0	8.411	4.916	2	8.223	2.525	3
950	8.652	15.443	1	8.552	4.93	2	8.678	2.519	3
1000	8.285	12.399	1	8.371	4.916	2	8.375	2.616	3
1600	7.846	6.074	1	7.927	4.915	2	7.872	2.529	3
1650	8.918	2.691	3	8.878	4.904	2	8.854	2.523	4
1700	7.839	6.506	1	7.697	4.897	2	7.8	2.522	3
1750	6.252	30.173	0	6.523	5.09	1	6.399	2.526	3
1800	8.643	26.655	0	8.218	4.918	2	8.143	2.487	3
1850	8.072	2.901	3	8.026	4.901	2	8.095	2.532	3
1900	8.442	30.468	0	8.495	4.927	2	8.297	2.516	3
1950	8.123	2.542	3	8.367	4.927	2	7.951	2.495	3
2000	8.366	12.175	1	8.58	5.13	2	8.394	2.533	3
